# A *Prunus*-specific small peptide suppresses anthocyanin accumulation by targeting HYH-BBX signaling in peach

**DOI:** 10.1186/s43897-026-00274-3

**Published:** 2026-09-09

**Authors:** Lei Zhao, Liao Liao, Weihan Zhang, Juanli Sun, Di Ai, Jian-Ping An, Yuepeng Han

**Affiliations:** 1 State Key Laboratory of Plant Diversity and Specialty Crops, Wuhan Botanical Garden, Chinese Academy of Sciences, Wuhan, 430074 China; 2 Hubei Hongshan Laboratory, Wuhan, 430070 China; 3 University of Chinese Academy of Sciences, 19 A Yuquanlu, Beijing, 100049 China

The blood-fleshed peach has gained significant consumer interest due to its rich composition of anthocyanins which offer significant health benefits, such as cardiovascular disease prevention and anti-cancer properties (Zhang 2021). The blood flesh of the peach is a Mendelian trait, but its inheritance pattern can be dominant or recessive. The dominant blood-flesh trait that is characterized by anthocyanin accumulation during the late stages of development is controlled by the *BLOOD* (*BL*) gene on chromosome (Chr) 5 (Zhou et al. 2015; Hara-Kitagawa et al. 2020), whereas the recessive blood-flesh trait is determined by a single recessive locus (*bf*) that was mapped to the top of Chr4 spanning from 0 to 1.38 Mb (Gillen and Bliss 2005; Rahim et al. 2014). The recessive blood-flesh trait is characterized by initial pigmentation occurring around the pit hardening stage, intensifying until ripening, and accompanied by the red midrib leaf. The mechanism by which the *bf* locus controls the recessive blood-flesh trait remains unclear.

To investigate the mechanism of the recessive blood-flesh trait, we compared the expression profiles of anthocyanin-related genes between the recessive blood-flesh peach ‘Heitao (HT)’ and the white-flesh peach ‘Baifeng (BF)’ (Methods S1). The flesh of ‘HT’ was green-colored at 30 days after flowering (DAF), started anthocyanin pigmentation at 45 DAF and intensified at 60 DAF, while the flesh of ‘BF’ was green-colored and had no anthocyanin accumulation until 60 DAF (Fig. 1A). Quantitative real-time PCR (qRT-PCR) analysis showed that anthocyanin structural genes and three MYB regulatory genes (*PpMYB10.1—PpMYB10.3*) had much higher levels of expression in ‘HT’ at 45 and 60 DAF than in ‘BF’ (Figure S1). Interestingly, the previously reported light-responsive *PpHYH*, which has two alternative splicing variants *PpHYH-X1* and *PpHYH-X2* (Zhao et al. 2022), showed strong up-regulation in ‘HT’, but had nearly no expression in ‘BF’ (Figure S2). These results suggest that anthocyanin accumulation in ‘HT’ is associated with activation of *PpMYB10.1-PpMYB10.3* and their upstream regulatory gene *PpHYH*, similar to the case of light-induced anthocyanin accumulation in fruit peel (Zhao et al. 2022). This finding prompted us to explore whether anthocyanin accumulation in ‘HT’ is light-dependent. The fruits of ‘HT’ were bagged at 30 DAF, and sampled at 60 DAF. Bagging treatment significantly inhibited flesh pigmentation and anthocyanin accumulation (Figure S3A). Consistently, the expression levels of *PpMYB10.1*, *PpHYH* and anthocyanin structural genes were dramatically down-regulated (Figure S3B). However, the expression of *PpHY5*, the key regulator of anthocyanin coloration in the peach flesh surrounding the stone (Zhao et al. 2023), was up-regulated under bagging treatment, suggesting that it is unrelated to the recessive blood-flesh trait. *PpHY5* is also non-essential for peach skin coloration (Zhao et al. 2022). PpHYH acts as a heterodimer with PpBBX4 to activate the transcription of *PpMYB10*, leading to peach skin coloration (Zhao et al. 2022). Thus, we addressed the regulatory roles of PpHYH and PpBBX4 in the flesh pigmentation in ‘HT’ using virus-induced gene silencing (VIGS) assay. Transient silencing of *PpHYH* or *PpBBX4* in the fruits of ‘HT’ caused down-regulation of the expression of anthocyanin structural and regulatory genes, resulting in a significant reduction in anthocyanin accumulation (Figure S4). This suggests that the PpHYH-PpBBX4 complex is essential in the control of the recessive blood flesh, similar to the mechanism observed in peach peel coloration.

**Fig. 1:**
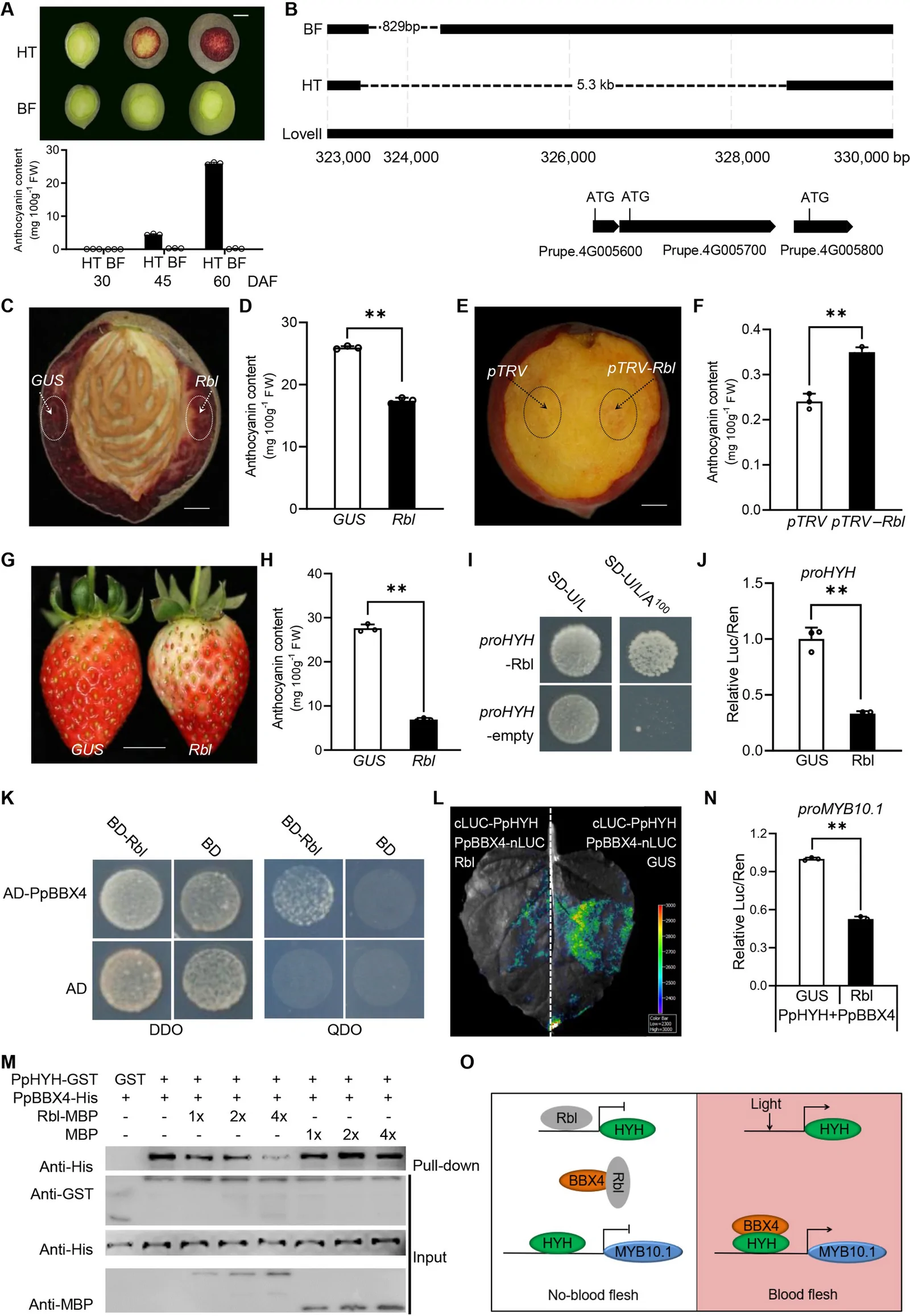
Analysis of the regulatory role of *Rbl* in anthocyanin accumulation. **A** Schematic diagram showing the pattern of anthocyanin accumulation in the fruits of ‘HT’ and ‘BF’ at 30, 45, and 60 days after flowering (DAF). The scale bar represents 1 cm. **B** Sequence variation in the genomic region spanning from 323,000 bp to 330,000 bp on chromosome 4 between ‘HT’ and ‘BF’. **C** Functional analysis of *Rbl* by its transient overexpression in the fruits of ‘HT’ at the mature stage. The *GUS* gene was used as the control, and the scale bar represents 1 cm. The white arrows indicate the infiltration sites and the sampled flesh tissues surrounding the infiltration site are highlighted using white dashed circle. **D** Influence of *Rbl* transient overexpression on anthocyanin accumulation in the fruits of ‘HT’. **E** Functional analysis of *Rbl* by its transient silencing in the fruits of ‘XHJ’ at the mature stage. The pTRV vector was used as the control, and the scale bar represents 1 cm. The black arrows indicate the infiltration sites and the sampled flesh tissues surrounding the infiltration site are highlighted using black dashed circle. **F** Influence of *Rbl* transient silencing on anthocyanin accumulation in the fruits of ‘XHJ’. **G** Functional analysis of *Rbl* through its transient overexpression in strawberry fruits. *GUS* was used as the control, and the scale bar represents 1 cm. **H** Influence of *Rbl* transient overexpression on anthocyanin accumulation in strawberry fruits. **I** The Y1H assay showing the capacity of Rbl to bind the *PpHYH* promoter. **J** The dual-luciferase reporter assays showing the activation activity of Rbl on the *PpHYH* promoter. **K** The Y2H assay showing the interaction between Rbl and PpBBX4. **L** The luciferase complementation assay showing the impact of Rbl on the formation of the PpBBX4-PpHYH complex. **M** The GST pull-down assay showing the influence of Rbl on the formation of the PpHYH-PpBBX4 complex. **N** The dual-luciferase reporter assay showing that Rbl impairs the activation activity of the PpHYH-PpBBX4 complex on the *PpMYB10.1* promoter. **O** A proposed model for the regulatory roles of Rbl in anthocyanin accumulation in peach. In non-blood flesh, Rbl represses the transcription of *PpHYH* and prevents the formation of the PpHYH-PpBBX4 complex, causing the failure of anthocyanin accumulation. Conversely, in recessive blood flesh, light-induced PpHYH can form a complex with PpBBX4 to induce transcription of *PpMYB10* due to the absence of the *Rbl* gene, thereby leading to anthocyanin pigmentation. The error bars in A, D, F, H, L and P represent the standard deviations of three biological replicates. The significance analysis in D, F, H, L and P was employed using the Student's *t*-test. ** indicates *P* < 0.01

To identify candidate genes for the *bf* locus, we compared the transcriptome profiles of fruits at 45 and 60 DAF between ‘HT’ and ‘BF’. Given that the *bf* locus is inherited in a recessive manner, we focused on the differentially expressed genes (DEGs) that showed down-regulation in ‘HT’ and up-regulation in ‘BF’. As a result, a total of 12 DEGs within the *bf* locus were identified (Table S1). Moreover, we sequenced the genomes of ‘HT’ and ‘BF’ to identify DNA sequence variations in the *bf* locus. The whole genome sequencing data were aligned to the peach reference genome v2.0 (Initiative et al. 2013). A deletion of 5.3-kb fragment (323,527 – 328,828 bp) within the *bf* locus was detected in the genome of ‘HT’, but with an 829-bp fragment deletion (323,527 – 324,355 bp) in ‘BF’ (Fig. 1B). Two out of the above-mentioned 12 DEGs, *Prupe.4G005700* and *Prupe.4G005800*, are located within or close to the 5.3-kb fragment, respectively. The expression profiles of these two genes were analyzed based on our previously reported fruit transcriptome database for four white-fleshed peach cultivars (Zheng et al. 2021). Both *Prupe.4G005700* and *Prupe.4G005800* had higher levels of expression in the four white-fleshed cultivars than in ‘HT’ (Figure S5). Transient transformation assays showed that overexpression of *Prupe.4G005800* in the fruits of ‘HT’ at 50 DAF caused a significant decrease in anthocyanin accumulation due to down-regulation of anthocyanin structural and regulatory genes (Fig. 1C,D; Figure S6). However, overexpression of *Prupe.4G005700* had little influence on anthocyanin accumulation and the expression of anthocyanin structural and regulatory genes (Figure S7). Thus, *Prupe.4G005800* was deemed as the candidate genes for the *bf* locus and designated *recessive blood* (*Rbl*). Notably, a photosynthesis-related gene *PpPSAK* in a candidate region of approximately 80 kb spanning from 4.32 to 4.40 Mb has been recently identified as a candidate gene for the *bf* locus because a 21-bp deletion in its coding sequence shows an association with the recessive red-flesh trait (Heurtevin et al. 2025). However, the 21-bp deletion did not occur in the *PpPSAK* gene in ‘HT’ (Figure S8), and its expression was higher in the 'HT' fruits at 45 and 60 DAF than that in ‘BF’ (Figure S9). Additionally, three DEGs between ‘HT’ and ‘BF’ were located in the region spanning from 1.38 to 4.40 Mb (Table S2). However, no structural variations were detected in these DEGs. These results supported *Rbl* as a candidate gene for the recessive blood-flesh trait.

The *Rbl* gene encodes a peptide of 94 amino acids (Figure S10). The amino acid sequences of *Rbl* were blasted against the NCBI database, and the *Rbl* homologs were found only in three other *Prunus* species, e.g. Taiwan cherry (*P. campanulata*), almond (*P. dulcis*), and Japanese apricot (*P. mume*). Thus, the *Rbl* gene seems to be unique to *Prunus* species. To validate the regulatory role of *Rbl* in anthocyanin accumulation, we conducted transient silencing assay in the fruits of peach variety ‘Xiahuangjin (XHJ)’. Silencing of *Rbl* caused a significant increase in anthocyanin accumulation (Fig. 1E,F). Consistently, the expression levels of anthocyanin-related genes were up-regulated (Figure S11). Moreover, ectopic transient overexpression of *Rbl* in strawberry fruits significantly inhibited anthocyanin accumulation (Fig. 1G,H), and the expression levels of anthocyanin-related genes were down-regulated (Figure S12). Finally, ectopic overexpression of *Rbl* in *Arabidopsis* significantly downregulated expression of anthocyanin-related genes in basal rosette leaves, resulting in a significant decrease in anthocyanin accumulation (Figure S13). Collectively, these results suggest that *Rbl* acts as a negative regulator of anthocyanin pigmentation. Since *BL* was not expressed in recessive blood-fleshed peach, *Rbl* and *BL* are likely to behave independently.

Subcellular localization assays showed that the Rbl protein was localized both in nucleus and cytoplasm (Figure S14). Yeast transformation assays showed that Rbl could attenuate the transcriptional activation activity of the GAL4-VP16 protein when it was fused with the VP16 transcriptional activation domain (Figure S15), suggesting that Rbl functions as a transcriptional repressor. To validate this hypothesis, we investigated whether Rbl could repress the transcription of *PpHYH* and *PpMYB10.1* which were the key regulators of anthocyanin accumulation in ‘HT’. Yeast one-hybrid (Y1H) and dual-luciferase reporter assays demonstrated that Rbl could bind to the promoter of *PpHYH* to repress its transcription (Fig. 1I,J). Likewise, Rbl could bind to the promoter of *PpMYB10.1* to suppress transcription (Figure S16). Notably, the inhibitory activity of Rbl is light-dependent (Figure S17A). DNA affinity purification sequencing (DAP‑seq) revealed a C/ACGT core motif for Rbl binding (Figure S18A). Three out of the five putative C/ACGT motifs in the *PpHYH* promoter were selected to design probes for electrophoretic mobility shift assays (EMSA) (Figure S18B). The results showed that Rbl bound specifically to the two C/ACGT motifs proximal to the ATG start codon (Figure S18C), and the binding capacity was abolished when the C/ACGT core motifs were mutated to TTTT (Figure S18D). Dual‑luciferase reporter assays indicated that Rbl had less repression activity on the mutated *PpHYH* promoter compared to the original promoter (Figure S18E). These results suggest that Rbl binds to the C/ACGT motif in promoter of *PpHYH* to suppress its transcription. The C/ACGT motif was also present in the promoter sequences of *FaMYB10* and *FaHY5* in strawberry. Thus, Rbl may bind to their promoters to repress transcription, leading to decreased anthocyanin pigmentation.

Unexpectedly, yeast two-hybrid (Y2H) assays indicated that Rbl was able to interact with PpBBX4 (Fig. 1K). Luciferase complementation and GST pull-down assays demonstrated that Rbl competed for binding to PpBBX4 with PpHYH (Fig. 1L,M). Dual-luciferase reporter assays showed that co‑infiltration of PpHYH and PpBBX4 strongly activated the *PpMYB10.1* promoter, but this activation was markedly reduced when *Rbl* was co-infiltrated (Fig. 1N). These results suggest that *Rbl* can repress anthocyanin biosynthesis by inhibiting the formation of the PpHYH‑PpBBX4 transcriptional complex. The previously reported candidate gene *PpPSAK* for the *bf* locus is assumed to interact with PpHY5 and PpMYB10 to activate the photosystem I complex, thereby affecting anthocyanin accumulation (Heurtevin et al. 2025). Therefore, *PpPSAK* and *Rbl* may regulate flesh coloration by different mechanisms.

Plant small peptides have been found to function in various aspects of growth and development (Zhang et al. 2025), but their roles in anthocyanin biosynthesis remain unclear. To our knowledge, we report for the first time the regulatory role of small peptides in fruit anthocyanin accumulation. In non-blood-fleshed peach fruits, *Rbl* represses anthocyanin accumulation by suppressing transcription of *PpHYH* and *PpMYB10*.*1* as well as inhibiting the formation of the PpHYH-PpBBX4 complex (Fig. 1O). Conversely, in recessive blood-fleshed peach fruits, the absence of *Rbl* allows PpHYH and PpBBX4 to form a complex to activate the transcription of *PpMYB10*.*1*, leading to anthocyanin pigmentation. Notably, unlike *PpHYH* that is light responsive, *Rbl* was not sensitive to light signals at the transcriptional level (Figure S17B). It has been reported that light signaling induces the formation of the PpHYH-PpBBX4 complex that induces the transcription of *PpMYB10.1* to promote anthocyanin pigmentation in peach peel (Zhao et al. 2022). A similar mechanism is likely responsible for light-dependent anthocyanin accumulation in the recessive blood-flesh peach. In pear, BBX TFs form a heterodimer with HY5 to regulate transcription of MYB10, which is responsible for light-induced anthocyanin pigmentation in the peel (Bai et al. 2019). Thus, the HY5/HYH-BBX complex seems to act as key player in the regulation of light-induced anthocyanin pigmentation in the fruits of Rosaceae.

## Supplementary Information

Supplementary Material 1.

## Data Availability

All data are available in the manuscript.
