# Supplementary material for: A Prunus-specific small peptide suppresses anthocyanin accumulation by targeting HYH-BBX signaling in peach
Source: Mol Hortic. 2026 Sep 9;6:77. doi: 10.1186/s43897-026-00274-3 (PMC13555937; doi:10.1186/s43897-026-00274-3)
Supplement: Supplementary file 1 [file 43897_2026_274_MOESM1_ESM.docx]

Supporting Information

Article title: A small peptide Rbl represses anthocyanin accumulation in peach

Lei Zhao^1,2^, Liao Liao^1,2^, Weihan Zhang^1,2^, Juanli Sun^1,3^, Di Ai^1,3^, Jian-Ping An^1,2*^, Yuepeng Han^1,2*^

The following Supporting Information is available for this article.

**Figure S1** Expression profiles of anthocyanin structural and regulatory genes in flesh tissues of ‘HT’ and ‘BF’ at 30, 45, and 60 DAF. The error bars represent the ±SD of three biological replicates.

**Figure S2** Expression profiles of two alternative splicing variants of *PpHYH* in flesh tissues of ‘HT’ and ‘BF’ at 30, 45, and 60 DAF. The error bars represent the ±SD of three biological replicates.

**Figure S3** Effect of bagging treatment on anthocyanin pigmentation and anthocyanin-related gene expression in the flesh of ‘HT’. (A) Effect of bagging treatment on anthocyanin pigmentation and anthocyanin accumulation in the flesh of ‘HT’. (B) Effect of bagging treatment on the expression of anthocyanin structural regulatory genes in the flesh of ‘HT’. The error bars represent the ±SD of three biological replicates and statistical significance was determined using the Student’s *t*-test. **, *P* < 0.01.

**Figure S4** Functional analysis of *PpHYH* and *PpBBX4* through their transient silencing in the fruits of ‘HT’ at the mature stage. (A) Schematic diagram showing the assay of *PpHYH* transient silencing using the VIGS system. The white arrows indicate the infiltration sites and the sampled flesh tissues surrounding the infiltration site are highlighted using white dashed circle. (B) Influence of *PpHYH* silencing on anthocyanin accumulation. (C) Influence of *PpHYH* silencing on the expression of anthocyanin-related genes. (D) Transient silencing of *PpBBX4* using the VIGS system. The white arrows indicate the infiltration sites and flesh tissues surrounding the infiltration site are highlighted using white dashed circle. (E) Influence of *PpBBX4* silencing on anthocyanin accumulation. (F) Influence of *PpBBX4* silencing on the expression of anthocyanin-related genes. The error bars represent the ±SD of three biological replicates and statistical significance was determined using the Student’s *t*-test. **, *P* < 0.01.

**Figure S5** The expression heatmap of *Prupe.4G005700* and *Prupe.4G005800* in the flesh tissues of six peach varieties at three developmental stages. The suffixes, -1, -2 and -3, represent 30, 45, and 60 DAF, respectively. HT, Heitao; BF, Baifeng, LGLT, Ligelante; MGWY, Meiguowanyou; XiaCui (XC), and XH, Xiahui.

**Figure S6** Functional analysis of *Rbl* through its transient overexpression in the fruits of ‘HT’ at the mature stage. (A) The expression of *Rbl* in the flesh tissues infiltrated with *Rbl* and *Gus* (control). (B) The expression of anthocyanin-related genes in flesh tissues infiltrated with *Rbl* and *Gus* (control). The error bars represent the ±SD of three biological replicates and statistical significance was determined using the Student’s *t*-test. **, *P* < 0.01.

**Figure S7** Functional analysis of *Prupe.4G005700* through its transient overexpression in the fruits of ‘HT’ at the mature stage. (A) Schematic diagram showing the transient overexpression assay for *PpHYH*. The white arrows indicate the infiltration sites and the sampled flesh tissues surrounding the infiltration site are highlighted using white dashed circle. *GUS* was used as the negative control. (B) The expression of *Prupe.4G005700* in the flesh tissues infiltrated with *Prupe.4G005700* and *GUS*. (C) Anthocyanin content in the flesh tissues infiltrated with *Prupe.4G005700* and *GUS*. (D) The expression of anthocyanin-related genes in the flesh tissues infiltrated with *Prupe.4G005700* and *GUS*. The error bars represent the ±SD of three biological replicates and statistical significance was determined using the Student’s *t*-test. ns, not significant.

**Figure S8** Detection of the 21-bp fragment associated with *PpPASK* in ‘HT’ using PCR based sequencing method. The 21-bp fragment whose deletion is associated with the *bf* trait is highlighted in blue background.

**Figure S9** Analysis of the expression of *PpPSAK* in the fruits of ‘HT’ and ‘BF’ at 45, and 60 DAF. The error bars represent the ±SD of three biological replicates.

**Figure S10** Genomic structure and amino acid sequences of the *Rbl* gene.

**Figure S11** Functional analysis of *Rbl* using the VIGS system. (A) The *Rbl* expression in the flesh tissues of the mature fruits of ‘XHJ’ with *Rbl* silenced or pTRV infiltration (control). (B) The expression of anthocyanin-related genes in the flesh tissues of the mature fruits of ‘XHJ’ with *Rbl* silenced or pTRV infiltration (control).

**Figure 12** Functional analysis of *Rbl* through its transient overexpression in strawberry fruits. (A) The expression of *Rbl* in the fruits infiltrated with *Rbl* or *GUS* (control). (B) The expression of anthocyanin-related genes in strawberry fruits infiltrated with *Rbl* or *GUS* (control). The error bars represent the ±SD of three biological replicates and statistical significance was determined using the Student’s *t*-test. **, *P* < 0.01.

**Figure S13** Functional analysis of *Rbl* through its ectopic overexpression in *Arabidopsis*. (A) Difference in anthocyanin pigmentation in basal rosette leaves between WT and transgenic lines overexpressing *Rbl*. Sample tissues collected for qRT-PCR analysis and anthocyanin measurement are indicated by white circles. The scale bar represents 1 cm. (B) The expression of *Rbl* in wild type (WT) and *Rbl* overexpression lines. (C) Influence of *Rbl* stable overexpression on anthocyanin accumulation in basal rosette leaves. (D) The expression of anthocyanin-related genes in basal rosette leaves of WT and transgenic lines. The error bars represent the ±SD of three biological replicates and statistical significance was estimated using the Student’s *t*-test. **, *P* < 0.01.

**Figure 14** Subcellular localization analysis of the Rbl protein

**Figure S15** Assessment of the repression activity of the *Rbl* gene using the GAL4-VP16 transactivation system. (A) Schematic diagram showing the Rbl protein fused with the VP16 transcriptional activation domain. (B) Yeast hybridization assays showing the transcriptional activity of the Rbl protein.

**Figure S16** Assessment of the repressive effect of Rbl on the *PpMYB10.1* promoter. (A) Y1H showing the binding capacity of Rbl to the *PpMYB10.1* promoter. (B) The dual-luciferase reporter assay showing the repressive effect of Rbl on the *PpMYB10.1* promoter. The error bars represent the ±SD of three biological replicates and statistical significance was determined using the Student’s *t*-test. **, *P* < 0.01.

**Figure S17** Analysis of the influence of shading on *Rbl*-mediated repression and *Rbl* transcription. (A) Dual-luciferase reporter assays showing the influence of shading on *Rbl*-mediated transcriptional regulation of the *PpHYH* and *PpMYB10.1* promoters. (B) Analysis of the influence of shading on *Rbl* transcription using dual-luciferase reporter assay. The error bars represent the ±SD of three biological replicates and statistical significance was determined using the Student’s *t*-test. **, *P* < 0.01.

**Figure S18** Assessment of Rbl repression activity on the *PpHYH* promoter. (A) Identification of Rbl binding sites using DAP-seq. (B) Schematic diagram showing the C/ACGT motifs in the *PpHYH* promoter region. P1-P3 and P2M/P3M are the probes used for EMSA, and the core motif sequences or their mutated sequences are highlighted in black bold. (C) EMSA showing that Rbl specifically binds to the probes containing the C/ACGT motif proximal to the start codon, e.g. P2 and P3. (D) Validation of the binding ability of Rbl to the C/ACGT motif using EMSA. (E) Evaluation of transcriptional activity of Rbl on the *PpHYH* promoter and its mutated versions using dual‑luciferase reporter assays. The error bars represent the ±SD of three biological replicates. Different lowercase letters indicate significant differences at *P* < 0.05 as determined by Fisher’s least significant difference (LSD) test.

**Table S1** DEGs showed down-regulation in the fruits of ‘HT’ at 30, 45, and 60 DAF, but with up-regulation in ‘BF’

| Accession number | 30 DAF | | | | | | 45 DAF | | | | | | 60 DAF | | | | | | Annotation |
| --- | --- | --- | --- | --- | --- | --- | --- | --- | --- | --- | --- | --- | --- | --- | --- | --- | --- | --- | --- |
|  | HT1_1 | HT1_2 | HT1_3 | BF1_1 | BF1_2 | BF1_3 | HT2_1 | HT2_2 | HT2_3 | BF2_1 | BF2_2 | BF2_3 | HT3_1 | HT3_2 | HT3_3 | BF3_1 | BF3_2 | BF3_3 |  |
| Prupe.4G005700 | 0.00 | 0.00 | 0.00 | 45.45 | 39.66 | 57.34 | 0.00 | 0.00 | 0.00 | 85.03 | 76.46 | 79.18 | 0.00 | 0.00 | 0.00 | 69.68 | 77.85 | 48.30 | N/A |
| Prupe.4G005800 | 0.18 | 0.22 | 0.10 | 18.67 | 23.68 | 37.64 | 1.27 | 0.31 | 0.00 | 28.74 | 33.40 | 27.00 | 0.14 | 0.10 | 0.18 | 13.81 | 21.08 | 19.29 | N/A |
| Prupe.4G011500 | 0.01 | 0.02 | 0.00 | 6.28 | 5.50 | 6.51 | 0.03 | 0.04 | 0.00 | 5.05 | 4.86 | 5.74 | 0.00 | 0.00 | 0.00 | 6.03 | 8.12 | 6.81 | Vacuolar protein sorting-associated protein 27 |
| Prupe.4G017700 | 131.21 | 159.00 | 257.27 | 23.22 | 60.87 | 96.54 | 8.34 | 18.23 | 12.23 | 74.86 | 77.86 | 66.97 | 19.09 | 18.73 | 14.09 | 116.70 | 104.08 | 89.57 | Aspartic proteinase |
| Prupe.4G019700 | 12.18 | 18.43 | 19.83 | 29.27 | 29.40 | 27.96 | 5.74 | 3.54 | 2.56 | 15.44 | 18.69 | 18.99 | 0.86 | 0.77 | 0.00 | 10.79 | 15.22 | 8.13 | N/A |
| Prupe.4G021100 | 25.19 | 22.28 | 24.27 | 275.91 | 121.63 | 51.83 | 364.89 | 359.73 | 268.62 | 2482.35 | 3144.51 | 2113.80 | 161.16 | 151.00 | 105.23 | 1115.03 | 1986.67 | 2139.27 | Peroxidase 42 |
| Prupe.4G025600 | 0.24 | 0.45 | 0.96 | 17.76 | 17.81 | 28.06 | 0.55 | 0.58 | 0.21 | 39.32 | 37.56 | 28.18 | 0.10 | 0.44 | 0.59 | 69.01 | 70.87 | 66.67 | Probable xyloglucan endotransglucosylase/hydrolase protein 8 |
| Prupe.4G026500 | 6.90 | 6.80 | 10.90 | 11.75 | 25.15 | 33.55 | 0.78 | 0.49 | 0.17 | 4.94 | 3.87 | 3.52 | 1.40 | 0.90 | 2.11 | 13.04 | 10.33 | 6.95 | N/A |
| Prupe.4G026600 | 15.04 | 20.56 | 30.58 | 26.26 | 45.71 | 58.26 | 0.55 | 2.52 | 1.26 | 9.73 | 12.05 | 10.97 | 6.20 | 4.30 | 5.05 | 37.21 | 24.60 | 23.01 | N/A |
| Prupe.4G028300 | 1.02 | 2.55 | 0.12 | 2.31 | 2.47 | 6.00 | 0.00 | 0.00 | 0.00 | 1.56 | 1.47 | 0.64 | 0.00 | 0.00 | 0.00 | 3.02 | 1.25 | 1.37 | Cysteine-rich receptor-like protein kinase 29 |
| Prupe.4G028500 | 0.00 | 0.00 | 0.00 | 1.77 | 0.99 | 0.70 | 0.00 | 0.00 | 0.00 | 1.97 | 1.35 | 0.59 | 0.00 | 0.00 | 0.00 | 0.46 | 1.46 | 1.15 | N/A |
| Prupe.4G030400 | 0.00 | 0.06 | 0.08 | 0.00 | 0.00 | 0.12 | 0.30 | 0.03 | 0.09 | 1.39 | 0.87 | 1.56 | 0.06 | 0.29 | 0.21 | 14.58 | 16.99 | 31.83 | (3S,6E)-nerolidol synthase 2, chloroplastic/mitochondrial |

**Table S2** DEGs showed down-regulation in the fruits of ‘HT’ at 30, 45, and 60 DAF, but with up-regulation in ‘BF’

| Accession number | 30 DAF | | | | | | 45 DAF | | | | | | 60 DAF | | | | | | Annotation |
| --- | --- | --- | --- | --- | --- | --- | --- | --- | --- | --- | --- | --- | --- | --- | --- | --- | --- | --- | --- |
|  | HT1_1 | HT1_2 | HT1_3 | BF1_1 | BF1_2 | BF1_3 | HT2_1 | HT2_2 | HT2_3 | BF2_1 | BF2_2 | BF2_3 | HT3_1 | HT3_2 | HT3_3 | BF3_1 | BF3_2 | BF3_3 |  |
| Prupe.4G051900 | 0.00 | 0.46 | 0.00 | 17.18 | 3.07 | 0.68 | 0.00 | 0.42 | 0.00 | 0.38 | 0.32 | 5.73 | 0.04 | 0.00 | 0.056 | 0.00 | 0.52 | 0.98 | N/A |
| Prupe.4G053300 | 7.90 | 16.22 | 30.56 | 5.94 | 6.32 | 11.40 | 1.51 | 3.35 | 3.26 | 15.41 | 15.31 | 15.50 | 0.86 | 1.31 | 0.44 | 29.01 | 18.50 | 21.97 | NAC domain-containing protein 73 |
| Prupe.4G079400 | 0.47 | 1.16 | 0.75 | 7.65 | 3.63 | 2.23 | 6.23 | 9.26 | 6.92 | 36.72 | 29.01 | 35.73 | 2.47 | 4.38 | 2.97 | 48.72 | 63.41 | 73.34 | Cytochrome P450 710A1 |

**Table S3** Primers for qRT-PCR analyses in peach

| **Name** | **Gene ID** | **Forward (5’→3’)** | **Reverse (5’→3’)** |
| --- | --- | --- | --- |
| PpCHS | Prupe.1G002900 | AACAAGGGTGCTCGTGTTCTC | GCTGCACCATCACCGAATAAG |
| PpCHI | Prupe.2G225200 | GAGATCGTTACAGGTCCATTTG | GTGGGAAGTTTTGATCCTTGA |
| PpDFR | Prupe.1G376400 | CGCCTCCAAGACTCTAGCTG | CCAGTGAGTGGGGAAAGTCC |
| PpLDOX | Prupe.5G086700 | AGGAGTTGAAGAAGGCAGCA | GCCTGGTCATTGGCATACTT |
| PpUFGT | Prupe.2G324700 | GTCCTAGCCCATGCTTCAGTC | GATCTCCAACACGTCCTCCAC |
| PpGST | Prupe.3G013600 | ACTTCAACTTCTGGTGCTGC | GCTGCTCTTTGATAATCTTTCC |
| PpMYB10.1 | Prupe.3G163100 | GAAATGATTGGTGGGAAACC | GTCCTTCTTCTGAAACATTGGT |
| PpMYB10.2 | Prupe.3G163000 | CACCATCAACAAGGATTGGA | CCGATTGTGGCATATCATCA |
| PpMYB10.3 | Prupe.3G163300 | AAGGCCACAACCAAGAAGA | AACCCAAGACCAGAACCTGT |
| PpbHLH3 | Prupe.8G242100 | TCTTGTTCAGCGTTCCGTTCCT | TTGGCGCTGAGCTCATCTTGTG |
| PpbHLH33 | Prupe.2G170100 | GGCCTGTGACATTGACGAAACTG | CTATAAGGGCATCTCATCTCTA |
| PpWD40 | Prupe.2G319500 | TTGGGACTTCCAGCATCGAC | AGATCCTCACGGACCCATCA |
| Ppactin | Prupe.8G132000 | TGCCATTGAAATCCTGAAAC | ACCAATTGGATCATCCTCCT |
| PpHYH-X1 | Prupe.1G208500 | AAGAGCAAACTAGACGTTCCTTGGT | TTCATCGCTCTCTTCTGGAACATT |
| PpHYH-X2 | Prupe.1G208500 | aaacaacaagaagagcaaactagaa | CGTTTTGCAGGAAAGCCAGAC |
| PpHY5 | Prupe.1G478400 | AATCTGCCGGAGCGTCAATCT | TTCAGCCTCTTGCTTTCCTTG |
| PpBBX4 | Prupe.4G156600 | tggatcttccaaggctgactg | tagagtcccgaggctgtagg |
| Rbl | Prupe.4G005800 | TGGTCGAAACACTGCAAGAGA | CCTTTGAAAGACAGAGCAAG |
| FvMYB10.1 | gene31413 | TCAAATCAGGCTTAAACAGA | TTAAAGACCACCTGTTTCCT |
| FvLDOX | gene32347 | GAAGTGCGTACCCAACTCCATCGT | ACCTTCTCCTTGTTGACGAGCCC |
| FvUFGT | gene12591 | CTAAGCAAAGGAAAGTTGAACGGAAT | TCCAACCGCAATGTGTTACAAA |
| Fv26S | gene11892 | TAACCGCATCAGGTCTCCAA | CTCGAGCAGTTCTCCGACAG |
| AtMYB75 | AT1G56650 | CAAGAGGTAGATATTTTGGTTCC | CTATACGCAAACGCAAACAAATG |
| AtLDOX | AT4G22880 | GTTTGCAGCTTTTCTACGAGG | TGAGCAAAAGTCCGTGGAGG |
| AtUFGT | AT5G54060 | TGTCAGATCGTTTTGGTTCC | GATTCTTCCTCACTTTCTCAC |
| Atactin | AT3G18780 | GCTGAGAGATTCAGATGCCCA | GTGGATTCCAGCAGCTTCCAT |

**Table S4** Summary of sequencing data quality

| **Sample name** | **Raw reads** | **Clean reads** | **Total mapped** | **Mapped ratio** | **Q20(%)** | **Q30(%)** | **GC content (%)** |
| --- | --- | --- | --- | --- | --- | --- | --- |
| HT1_1 | 50530200 | 49390260 | 46334913 | 93.81% | 97.31 | 92.78 | 46.26 |
| HT1_2 | 61023982 | 59838898 | 56333329 | 94.14% | 97.48 | 93.12 | 45.89 |
| HT1_3 | 47181798 | 45849154 | 43220192 | 94.27% | 97.48 | 93.13 | 45.93 |
| BF1_1 | 56852722 | 55752630 | 51844313 | 92.99% | 97.17 | 92.53 | 46.12 |
| BF1_2 | 62382750 | 61025146 | 57059984 | 93.50% | 97.29 | 92.78 | 45.7 |
| BF1_3 | 58375204 | 57095784 | 53791253 | 94.21% | 97.47 | 93.12 | 45.68 |
| HT2_1 | 52731002 | 51784084 | 48673466 | 93.99% | 97.37 | 92.94 | 46.68 |
| HT2_2 | 58193552 | 57072674 | 53496678 | 93.73% | 97.3 | 92.81 | 46.6 |
| HT2_3 | 49302082 | 48039416 | 44885247 | 93.43% | 97.23 | 92.65 | 46.87 |
| BF2_1 | 50127992 | 49017082 | 45680924 | 93.19% | 97.11 | 92.44 | 46.6 |
| BF2_2 | 51285040 | 49628428 | 46185400 | 93.06% | 97.19 | 92.61 | 46.99 |
| BF2_3 | 49254952 | 48345706 | 45283266 | 93.67% | 97.43 | 93.11 | 47.07 |
| HT3_1 | 62694082 | 61333192 | 57633605 | 93.97% | 97.33 | 92.82 | 46.83 |
| HT3_2 | 43957646 | 42951606 | 40562430 | 94.44% | 97.63 | 93.47 | 46.7 |
| HT3_3 | 46957540 | 45766386 | 43070498 | 94.11% | 97.26 | 92.71 | 46.82 |
| BF3_1 | 51868822 | 50960196 | 48212648 | 94.61% | 97.45 | 93.07 | 46.48 |
| BF3_2 | 46242528 | 45444590 | 42641732 | 93.83% | 97.36 | 92.91 | 46.54 |
| BF3_3 | 62734014 | 61725780 | 57944148 | 93.87% | 97.35 | 92.87 | 46.64 |

**Table S5** Primers for gene sequencing and vector construction

| Primer name | 5’→3’ | Enzyme site |
| --- | --- | --- |
| pSAK277-Rbl-F | actagtggatccaaagaattc ATGCCAGTGGTGGTCGAAAC | EcoRI |
| pSAK277-Rbl-R | gactctagaagtactctcgag TCATTTAATGAAGCTGTAATT | XhoI |
| pSAK277-PpHYH-F | gacactagtggatccaaaATGTCAGTCCCCATCAAACCAGG | EcoRI |
| pSAK277-PpHYH-R | gactctagaagtactTTACTTAACTGATCCTGCCTTTGGCT | XhoI |
| pSAK277-PpBBX4-F | ctagtggatccaaagaattc atgaagatacagtgcaacgt | EcoRI |
| pSAK277-PpBBX4-R | tcgagaagctttttgaattc ctagaattgcctacgacgtt | EcoRI |
| pTRV-PpHYH-F | gtgagtaaggttaccgaattc TACTTTCGTCCACTTTCGGCC | EcoRI |
| pTRV-PpHYH-R | cgtgagctcggtaccggatcc GAGAGCGATGAAGATATGTTC | BamHI |
| pTRV-PpBBX4-F | gtgagtaaggttaccgaattc tggatcttccaaggctgact | EcoRI |
| pTRV-PpBBX4-R | cgtgagctcggtaccggatcc tagagtcccgaggctgtagg | BamHI |
| pTRV-Rbl-F | gtgagtaaggttaccgaattc TGGTCGAAACACTGCAAGAG | EcoRI |
| pTRV-Rbl-R | cgtgagctcggtaccggatcc GGAGGTGCTTGAAGCCTTTG | BamHI |
| YFP-Rbl-F | TTACATTTACAATTAC ATGCCAGTGGTGGTCGAAAC | BamHI |
| YFP-Rbl-R | CCTCGCCCTTGCCCAT TTTAATGAAGCTGTAATT | BamHI |
| AD-Rbl-F | Gccatggaggccagtgaattc ATGCCAGTGGTGGTCGAAAC | EcoRI |
| AD-Rbl-R | agctcgagctcgatggatcc TCATTTAATGAAGCTGTAATT | BamHI |
| BD-Rbl-F | CATATGGCCATGGAGGCC atgccagtggtggtcgaaac | EcoRI |
| BD-Rbl-R | CGGCCGCTGCAGGTCGACG tcatttaatgaagctgtaat | BamHI |
| BD-VP16-Rbl-F | CTTTGACCTGGATGGATCC atgccagtggtggtcgaaac | EcoRI |
| BD-VP16-Rbl-R | GCTGCAGGTCGACGGATCC tcatttaatgaagctgtaat | BamHI |
| BD-PpBBX4-N-F | GCATATGGCCATGGAGGCC atgaagatacagtgcaacgt | EcoRI |
| BD-PpBBX4-N-R | CATATGGCCATGGAGGCC CTAGAGCAAAAACCTCCGGT | BamHI |
| pAbAi-PpHYH-F | AAGCTTGAATTCGAGCTC GGATGTACAGCATGCAAGCAC | KpnI |
| pAbAi-PpHYH-R | TACATACAGAGCACATGC GGCTTTAGCAGCTAGCTAGCTCT | XhoI |
| pAbAi-PpMYB10.1-F | AAGCTTGAATTCGAGCTC GATATCTGAAGACAGTTAAGCCGCG | KpnI |
| pAbAi-PpMYB10.1-R | TACATACAGAGCACATGC CTTCTTGTTGGCCAGCGTTGTAT | XhoI |
| pGreen-PpHYH-F | tatagggcgaattgggtacc ATTGTTGAATAGGAATTTGGA | KpnI |
| pGreen-PpHYH-R | atcgataccgtcgacctcgag GGCTTTAGCAGCTAGCTAGCT | XhoI |
| pGreen-PpMYB10.1-F | tatagggcgaattgggtacc GATATCTGAAGACAGTTAAGC | KpnI |
| pGreen-PpMYB10.1-R | atcgataccgtcgacctcgag CTTCTTGTTGGCCAGCGTTG | XhoI |
| pGreen-Rbl-F | tatagggcgaattgggtacc gaggctatttgctcttttta | KpnI |
| pGreen-Rbl-R | atcgataccgtcgacctcgag atttattcatttgttgctc | XhoI |
| cLUC-PpHYH-F | cccggggcggtacccgggat atgtcagtccccatcaaacca | BamHI |
| cLUC-PpHYH-R | gtagtccatttgttggatcc ttacttaactgatcctgcct | BamHI |
| nLUC-PpBBX4-F | acgagctcggtacccgggat atgaagatacagtgcaacgt | BamHI |
| nLUC-PpBBX4-R | gcgtacgagatctggtcgac gaattgcctacgacgttTTG | SalI |
| pGEX-PpHYH-F | AATCGGATCTGGTTCCGCGT atgtcagtccccatcaaacc | BamHI |
| pGEX-PpHYH-R | GGCCGCTCGAGTCGACCCGG ttacttaactgatcctgcct | BamHI |
| pET32a-PpBBX4-F | CCATGGCTGATATCGGATCC atgaagatacagtgcaacgt | EcoRI |
| pET32a-PpBBX4-R | CAAGCTTGTCGACGGAGCTC ctagaattgcctacgacgtt | EcoRI |
| pMAL-Rbl-F | AGGGAAGGATTTCAGAATTC ATGCCAGTGGTGGTCGAAAC | EcoRI |
| pMAL-Rbl-R | ACTCTAGAGGATCCGAATTC TCATTTAATGAAGCTGTAAT | EcoRI |

**Methods S1 Detailed description of materials and methods**

**Plant materials**

Fruit samples of the peach cultivars ‘Heitao (HT)’ and ‘Baifeng (BF)’ were collected at 30, 45, and 60 days after full bloom (DAF). The peach cultivar ‘Xiahuangjin (XHJ)’ was harvested during the early ripening stage. All peach cultivars were grown at the Wuhan Botanical Garden, Chinese Academy of Sciences (Wuhan, China). Upon harvest, the outer and inner pericarps were separated, and the inner pericarp tissue was immediately frozen in liquid nitrogen and stored at –80 °C until further analysis. Three biological replicates were collected for each sample. The strawberry cultivar ‘Hongyan’ was provided by the Institute of Economic Crops, Hubei Academy of Agricultural Sciences (Wuhan, China) and cultivated under natural light in a temperature-controlled greenhouse. Tobacco seedlings (*Nicotiana benthamiana*) were grown in a controlled-environment chamber under a 16 h/8 h day/night cycle at 25 °C.

**Determination of anthocyanin content**

Anthocyanin extraction and quantification were performed following our previously established method (Zhao *et al*., 2022). Approximately 200 mg of tissue was pulverized in liquid nitrogen, and the powder was immediately transferred into a 2‑mL centrifuge tube containing 1.5 mL of 0.1% hydrochloric acid‑methanol. Following 24 h incubation at 4 °C, the mixture was centrifuged at 13,400 × g for 20 min at room temperature. The supernatant was collected, transferred to a 10‑mL centrifuge tube, and diluted to a final volume of 5 mL with 0.1% hydrochloric acid‑methanol. Two 1‑mL aliquots of the extract were separately transferred into new 2‑mL centrifuge tubes, each containing 1 mL of either Buffer A (0.2 M KCl / 0.2 M HCl, 25:67, v/v, pH 1.0) or Buffer B (0.2 M NaAc, pH 4.5). After incubation at 4 °C for 2 h, absorbance was measured at 510 nm and 700 nm. The relative anthocyanin content was calculated as: TA = [(A_510_ − A_700_) pH 1.0 − (A_510_ − A_700_) pH 4.5] / g fresh weight (FW). Each sample contained three biological replicates.

**cDNA librariy constructions and sequencing**

The fruit samples of 'HT' and 'BF' at 30, 45 and 60 DAF were used for cDNA library construction and sequencing. Total RNA was extracted using the HiPure HP Plant RNA Mini Kit (Magen, Guangzhou, China) and cDNA libraries were prepared using the CloneMiner™ cDNA Library Construction Kit (Thermo Fisher Scientific, Waltham, USA). The libraries were sequenced on the Illumina HiSeq Xten platform by Beijing Nohe Zhiyuan Technology Co., Ltd. Raw reads were processed to remove low-quality sequences and adapters, and clean reads were aligned to the reference peach genome (Initiative *et al*., 2013). Gene expression levels were quantified as Fragments Per Kilobase of transcript per Million mapped reads (FPKM).

**Structural variations (SVs) detection**

Approximately 2 µg of high‑quality genomic DNA from 'HT' and 'BF' was prepared using the Ligation Sequencing Kit SQK‑LSK109 (Oxford Nanopore Technologies). DNA was fragmented to approximately 5 kb using G‑TUBE devices at 5,000 rpm, followed by damage repair and end‑dA‑tailing with NEBNext FFPE DNA Repair Mix and NEBNext Ultra II End Repair/dA‑Tailing Modul (New England Biolabs). Native barcodes and sequencing adapters were ligated with the Native Barcoding Expansion 1‑12 Kit (EXP‑NBD104, Oxford Nanopore Technologies). Libraries were sequenced on a PromethION flow cell (Oxford Nanopore Technologies). Raw reads were processed with custom Perl scripts to remove low‑quality sequences and adapters. Clean reads were aligned to the peach reference genome v2.0 and structural variants (SVs) were detected with Sniffles software.

**RNA extraction and quantitative real-time PCR (qRT-PCR) analysis**

Total RNA was extracted from each fruit sample and quantified using an ND-1000 UV spectrophotometer, with concentrations adjusted to 500 ng/μL. cDNA synthesis was performed with the PrimerScript™ RT Reagent Kit with gDNA Eraser (Yeasen, Shanghai, China). qRT‑PCR was carried out on a 7300 Real-Time PCR System (Applied Biosystems™, USA), following the reaction system and amplification program described previously (Zhao et al., 2022). The glyceraldehyde-3-phosphate dehydrogenase gene (*PpGADPH*) was used as an internal control. Gene expression levels were calculated using the comparative Ct (2^–ΔΔCT^) method.

**Subcellular localization assay**

The full-length coding sequence of *Rbl* was amplified using cDNA from ‘BF’ as the template and subsequently cloned into the pFGC-YFP vector via homologous recombination. The resulting construct was introduced into *Agrobacterium tumefaciens* strain GV3101. After culturing the bacterial suspension, it was infiltrated into the abaxial side of leaves from 4-week-old tobacco plants. Two days after infiltration, fluorescent signals were observed under a confocal microscope (TCS SP8, Leica Microsystems, Wetzlar, Germany).

**Y2H and Y1H assays**

For yeast transformation assay, the full-length coding sequence of *Rbl* was amplified and subsequently cloned into the BD-VP16 vector. The resulting construct was transformed into the yeast Y2H Gold strain. After three days of culture, transformations were plated onto both SD-Trp and SD-Trp + X-α-Gal + AbA plates for analysis.

For Y2H assay, the full-length coding sequence of *Rbl* and *PpBBX4* was amplified from the cDNA of ‘BF’ and cloned into the BD (pGBKT7) and AD (pGADT7) vectors, respectively. These constructs were then co-transformed into the yeast Y2H Gold strain. After three days of culture, transformants were plated onto DDO (SD/–Trp/–Leu) and QDO (SD/–Trp/–Leu/–His/–Ade) plates for analysis.

For Y1H assay, the full-length coding sequence of *Rbl* and was amplified and subsequently cloned into the AD vector. Approximately 2.0 kb promoter fragment of *PpHYH* or *PpMYB10.1* was amplified from 'HT' genomic DNA and cloned into the pAbAi vector. The recombinant constructs were co-transformed into the yeast strain Y1H Gold. After three days of growth, transformants were plated onto SD/–Ura and SD/–Ura plates supplemented with Aureobasidin A (AbA)* for selection, where * indicates the minimum inhibitory concentration of AbA. The AD-empty vector was used as a negative control.

**Firefly luciferase complementation assay**

The Firefly luciferase complementation assay was performed as previously described (Zhao et al., 2022). The full-length coding sequences of *PpHYH* and *PpBBX4* were amplified and cloned into the pCambia1300‑cLUC and pCambia1300‑nLUC vectors, respectively, while the *Rbl* coding sequence was inserted into the pSAK277 overexpression vector. All constructs were transformed into *A. tumefaciens* strain GV3101. Agrobacterium bacterial suspensions carrying the three constructs were mixed in a 1:1:1 ratio and co‑infiltrated into the abaxial side of tobacco leaves. Two days after infiltration, luminescence signals were detected using an ImageQuant LAS4000 mini chemiluminescence imaging system (GE Life Sciences).

**Dual-luciferase reporter assay**

The pSAK277-Rbl was constructed as described above, while the promoter sequences of *PpHYH* or *PpMYB10.1* was inserted into the pGreenII 0800-LUC vector. Both constructs were introduced into *A. tumefaciens* strain GV3101. Agrobacterium bacterial suspensions containing the two constructs were mixed at a 9:1 ratio and co-infiltrated into the abaxial side of tobacco leaves. Samples were collected two days post-infiltration, and dual- luciferase reporter assay was performed using the Dual Luciferase Reporter Gene Assay Kit (Promega) to measure firefly and Renilla luciferase activities. Results are expressed as the ratio of firefly to Renilla luminescence (Luc/Ren). Three biological replicates were conducted for each treatment.

**Electrophoretic mobility shift assay (EMSA)**

EMSA was performed to evaluate the binding of Rbl to the C/ACGT motifs in the *PpHYH* promoter. Biotin‑labeled DNA probes were incubated with purified recombinant Rbl protein in binding buffer for 20 min at room temperature. For competition assays, a 10- and 50-fold excess of unlabeled probe was added. Mutant probes with the C/ACGT core mutated to TTTT served as negative controls. Protein‑DNA complexes were resolved on a 6% native polyacrylamide gel in 0.5× TBE buffer at 4 °C. After electrophoresis, the gel was transferred to a nylon membrane, cross‑linked, and biotin‑labeled DNA was detected using the Chemiluminescent EMSA Kit (Beyotime, Shanghai, China). Signals were visualized with a chemiluminescence imaging system.

**GST pull-down assay**

The coding sequences of *PpHYH*, *PpBBX4*, and *Rbl* were individually cloned into the pGEX-4T, pET-32a, and pMAL-C2 vectors to generate the recombinant fusion constructs PpHYH-GST, PpBBX4-His, and Rbl-MBP. These constructs were transformed into *E. coli* BL21 for prokaryotic expression. Recombinant proteins were purified using ProteinIso GST Resin (TransGen Biotech, Beijing, China), Ni-NTA Resin (BeyoGold Biotech, Shanghai, China), and MBPSep Dextrin Agarose Resin (Yeasen, Shanghai, China), respectively. Depending on the experimental combinations, the proteins were mixed and incubated in 1 ml of binding buffer (50 mM Tris-HCl, pH 7.5, 100 mM NaCl, 0.25% v/v Triton X-100, and 35 mM β-mercaptoethanol). GST Resin was then added to adsorb the protein complexes, followed by elution with the binding buffer. Protein content was analyzed by western blot using anti-GST, anti-His, and anti-MBP antibodies, and signals were visualized with the FluorChem R System.
